# The Principles of Electroconvulsive Therapy Based on Correlations of Schizophrenia and Epilepsy: A View From Brain Networks

**DOI:** 10.3389/fneur.2019.00688

**Published:** 2019-06-27

**Authors:** Qi Li, Sha Liu, Meng Guo, Cheng-Xiang Yang, Yong Xu

**Affiliations:** ^1^Department of Psychiatry, First Hospital/First Clinical Medical College of Shanxi Medical University, Taiyuan, China; ^2^MDT Center for Cognitive Impairment and Sleep Disorders, First Hospital of Shanxi Medical University, Taiyuan, China; ^3^National Key Disciplines, Key Laboratory for Cellular Physiology of Ministry of Education, Department of Neurobiology, Shanxi Medical University, Taiyuan, China; ^4^Department of Humanities and Social Science, Shanxi Medical University, Taiyuan, China

**Keywords:** electroconvulsive therapy (ECT), schizophrenia, temporal lobe epilepsy (TLE), brain networks, graph theory

## Abstract

Electroconvulsive therapy (ECT) was established based on Meduna's hypothesis that there is an antagonism between schizophrenia and epilepsy, and that the induction of a seizure could alleviate the symptoms of schizophrenia. However, subsequent investigations of the mechanisms of ECT have largely ignored this originally established relationship between these two disorders. With the development of functional magnetic resonance imaging (fMRI), brain-network studies have demonstrated that schizophrenia and epilepsy share common dysfunctions in the default-mode network (DMN), saliency network (SN), dorsal-attention network (DAN), and central-executive network (CEN). Additionally, fMRI-defined brain networks have also been shown to be useful in the evaluation of the treatment efficacy of ECT. Here, we compared the ECT-induced changes in the pathological conditions between schizophrenia and epilepsy in order to offer further insight as to whether the mechanisms of ECT are truly based on antagonistic and/or affinitive relationships between these two disorders.

## Introduction

Electroconvulsive therapy (ECT) is one of the oldest therapeutic modalities in psychiatric clinical practice. The therapeutic effects of ECT putatively depend on the ECT-induced manifestation of seizure-like states, which was first proposed by the Hungarian neuropsychiatrist Ladislas Meduna in 1934 (1). This hypothesis arose from conspicuously opposite pathological results in epileptic vs. schizophrenic patients; Meduna observed an excess of glial cells in the brain tissue of epileptic patients (2), while his colleague found a reduction of glial cells in the brain tissue of schizophrenic patients (3). This findings convinced Meduna that there was an antagonism between schizophrenia and epilepsy, and gave birth to the idea that induction of seizure might help to alleviate the symptoms of schizophrenia. Subsequently, Meduna conducted the first human experiment by using camphor intramuscular injections (4). Finally, camphor was replaced by electricity to achieve more stable therapeutic effects by Ugo Cerletti and Lucio Bini, from which ECT was born (5).

Although Meduna' hypothesis has since been refuted by the finding that different glial subtypes have different pathological features and do not exhibit a homogeneous opposing relationship between epilepsy and schizophrenia (6, 7), the relationship between these two disorders is still an interesting topic and has been debated for many years (8, 9). Schizophrenia is a serious psychiatric disorder that is always characterized by positive symptoms (hallucinations and delusions), negative symptoms (emotional disorders and impaired motivations), and cognitive impairment (10). Temporal lobe epilepsy (TLE) is the most widely studied and specific subtype of epilepsy (11, 12). Since these two neuropsychiatric disorders always shared some common symptoms, such as psychosis (13), emotion recognition disorders (14), and cognitive impairment (15), TLE showed a tight relation with schizophrenia in clinical diagnosis. The primary point of controversy is whether these two disorders have biological antagonism or affinity with one another. Some recent neuropathological studies still supported the antagonistic hypothesis for decreased astrocyte numbers in schizophrenia (16, 17) and increased astrocyte numbers and size in epilepsy (7). However, increasing sources of data also indicate that these two disorders may share some similarity. One study reported that there were some overlapping etiological factors between epilepsy and schizophrenia based on a population-based family study (18). Another study reported that schizophrenia and epilepsy share common features at the genetic level (19). As such, the relationship between epilepsy and schizophrenia is still an open question and requires further exploration for its elucidation.

Originating from the putative relationship between epilepsy and schizophrenia, ECT is now widely used for depression, acute manic episodes, catatonia, and treatment-resistant schizophrenia (20). Over the past 80 years, numerous psychological, psychoanalytical, and biological theories have been built to posit the potential therapeutic mechanisms of ECT (5). The present putative mechanisms of ECT have been primarily focused on structural, functional, and compositional changes of the brain after ECT treatment (21). Among these phenomena, the roles of cerebral blood flow (22), the blood-brain barrier (23), neurotransmitters (24), and the immune system (25) during ECT have been investigated. However, the potential mechanisms of ECT based on the correlation of epilepsy and schizophrenia remain largely unknown.

With the development of structural magnetic resonance imaging (sMRI) and functional MRI (fMRI), both structural and functional neuroimaging studies have provided more information to help better understand the relationship between schizophrenia and epilepsy (11, 26). Based on neuroimaging data, brain networks have been defined as correlational networks between several related brain regions in resting or task conditions. Disrupted brain networks—including the default-mode network (DMN), dorsal-attention network (DAN), central-executive network (CEN), and saliency network (SN)—have been detected in both schizophrenic (27, 28) and epileptic patients (29–31). These shared brain networks may help to better understand the relationship between schizophrenia and epilepsy. These disease-disrupted brain networks can also be used as valuable biomarkers for assaying the therapeutic effects of ECT (32–34), which have shown apparent changes after ECT treatment at both structural and functional levels. Thus, comparison of the ECT-induced changes in the pathological conditions of schizophrenia and epilepsy may offer further insight as to whether the mechanisms of ECT are truly based on antagonistic and/or affinitive relationships between these two disorders.

This review will focus on brain-network changes in schizophrenia and epilepsy to discuss the affinity and/or antagonism between these two disorders. Our view was synthesized based on brain networks including the DMN, DAN, CEN, and SN, and a more large scale assessment of networks from graph theory. Additionally, changes in brain networks after ECT treatment will be compared under these two pathological conditions to help better understand the principles of ECT. All of the cited articles included in this review were written and published in English and were published before January 2019. The search engine, PubMed, was used with MESH terms.

## Default-Mode Network

**Figure 1:**
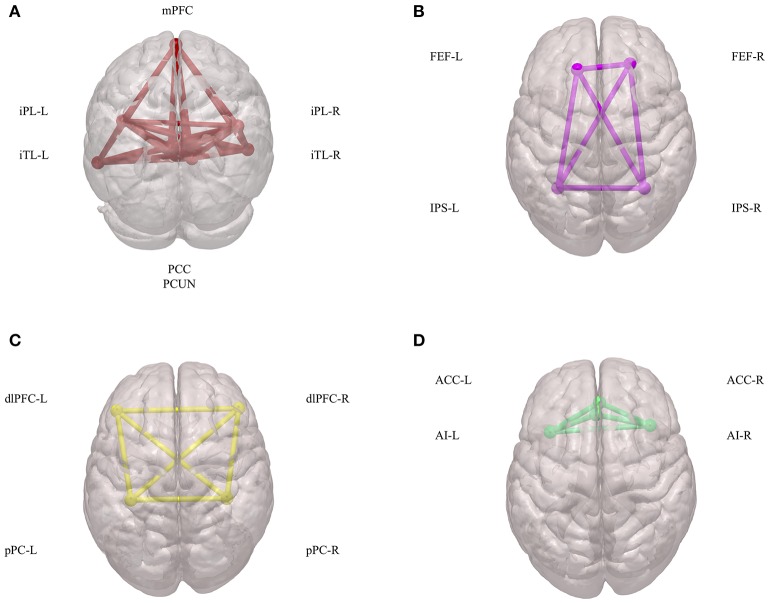
Components of DMN, DAN, CEN, and SN. **(A)** Spatial components of DMN. **(B)** Spatial components of DAN. **(C)** Spatial components of CEN. **(D)** Spatial components of SN. DMN, default mode network; DAN, dorsal attention network; CEN, central executive network; SN, salience network; mPFC, medial prefrontal cortex; iPL, inferior parietal lobe; iTL, inferior temporal lobes; PCC, posterior cingulate cortex; PCUN, adjacent precuneus; FEF, frontal eye fields; IPS, intraparietal sulcus; dlPFC, dorsal lateral prefrontal cortex; pPC, posterior parietal cortex; ACC, anterior cingulate cortex; AI, anterior insula.

Initial fMRI studies mainly focused on task-induced increases in regional brain activities during goal-directed behaviors. Subsequently, it was found that there are also consistent and task-independent decreases in regional brain activities, which is a phenomenon that has been defined as the baseline or default mode of brain function (35). Since then, the default mode of brain function has generated far more interest, discussion, and controversy, and it has called more attention to the importance of intrinsic brain activities (36). Combined with blood-oxygen-level-dependent (BOLD) signals and diffusion-tensor imaging (DTI) data, these intrinsic brain activities have been shown to depend on networks across several brain regions, which collectively has been termed the DMN and consists of the following: the posterior cingulate cortex (PCC); adjacent precuneus (PCUN); medial prefrontal cortex (mPFC); mesial and inferior temporal lobes (mTL/iTL); and the inferior parietal lobe (iPL) (37) (Figure 1A). Functionally, these intrinsic brain activities of the DMN are activated at rest, become deactivated at the initiation of a task, and play an important role in cognitive functions and emotional processing (38). In the N-back working-memory task, when cognition load increased, the functional connectivity within the DMN concomitantly decreased (39). Additionally, during long-term stabilization of memory, the intra-network synchrony of the DMN was positively corelated to individual performance (40). In emotional processing, less decreased DMN activity was highly related to poor emotionality (41). These physiological signatures of the DMN have attracted more and more attention in terms of their promising clinical applications in assessing and treating neuropsychiatric disorders.

Brain network studies have demonstrated increasing evidence of correlations between the DMN and some symptoms of schizophrenia (42). Auditory verbal hallucinations represent the most common positive symptom of schizophrenia and induce alterations of functional connectivity within the DMN (43). Additionally, patients with delusions also exhibit reduced regional deactivation of the DMN (44). Besides typical symptoms, positive symptoms scored by Positive and Negative Syndrome Scale (PANSS) have also been shown to be positively correlated with increased deactivation of brain regions, including the medial frontal, temporal, and cingulate gyri (45). For negative symptoms, emotional disorders of patients were found to be positively related to the magnitude of deactivation of rAC and mPFC regions (46). Additionally, negative symptoms scored by SANS have been shown to have a linear correlation to the functional connectivity within the DMN (47). These results indicate that changes of deactivation and functional connectivity within the DMN may represent a valuable means for assessing positive and negative schizophrenic symptoms; however, further evidence is required before this potential utility is sufficiently verified. Since the DMN is an important brain network that participates in cognitive processes, the relationship between the DMN and cognitive impairment of schizophrenia has also been widely studied. In a working memory task, the mPFC showed less deactivation (48) and greater activation (49). When the task load changed, DMN functions were over-recruited during a low-task load, and hyper-deactivated during a high-task load (50). Other brain regions, such as the left-superior temporal gyrus, have also been shown to be positively correlated to cognitive impairment (51). In addition to BOLD-based functional studies, DTI anatomical data have also revealed that altered frontal structural connectivity is corelated to cognitive ability as well as schizophrenic symptoms (52).

Studies of the DMN in TLE have indicated altered task-related deactivations compared with those of healthy controls (53) and reduced functional connectivity (54). However, it may be difficult to confirm the affinity or antagonistic relationship of psychiatric manifestations between schizophrenia and TLE based on the present DMN results. More well-designed investigations are needed in terms of elucidating the short-term and long-term effects of seizure on the DMN, and for the comparison of the symptoms of these two disorders. Similar to schizophrenia, the role of the DMN in TLE-induced cognitive impairment has also been comprehensively studied. In the N-back working-memory task, the ACC showed greater deactivation in TLE patients (55). Additionally, there was decreased functional connectivity between the mPFC and mTL/iTL (54), as well as the mPFC and hippocampus (56). Interestingly, comparison of the cognitive-related DMN changes between TLE and schizophrenia suggests an affinity between these two disorders. This relationship may be due to similar pathological changes in terms of the hippocampus exhibiting overlapping pathological features between the two disorders (57).

Clinical practices have demonstrated that ECT can effectively ameliorate the positive symptoms of schizophrenia (58) and significantly reduce PANSS scores (59). Additionally, a brain network study revealed that increased mTL connectivity and PCC volume were accompanied by clinical improvement (33). These results may suggest that the therapeutic effects of ECT are achieved by an opposite regulation of the DMN in schizophrenia. However, this hypothesis still requires additional evidence from large-sample investigations. Additionally, whether this opposing change of the DMN can be detected in TLE patients requires further investigation. By exploring seizure-induced changes in TLE patients, we may better elucidate the relationship between schizophrenia and TLE and any therapeutic outcomes of ECT. Although ECT is effective, the side effects of ECT still harbor great concerns (60). Also, although similar cognitive impairments between schizophrenia and TLE have been documented, further studies are required to determine whether the side effects of ECT are related to seizure-induced hippocampal dysfunction.

## Dorsal Attention Network

The DAN has been defined as a network that is comprised of the intraparietal sulcus (IPS) and the frontal eye fields (FEF) (61) (Figure 1B). Both IPS and FEF within the DAN play an important role in the maintenance of spatial attention, saccade planning, and visual working memory (62). The DAN is also activated during feature-based attention and provides a spatial coding in multiple reference frames (63). Furthermore, there is an interaction between the DAN and the DMN to carry out brain functions (64).

The DAN has been found to be altered in schizophrenic patients while carrying out several tasks that are mainly related to cognitive impairment. In visual attention and motor learning tasks, patients had reduced activation in the dorsal neocortical visual attention network (65). In a visual oddball task, connectivity between the right IPS (intraparietal sulcus) and right anterior insula (AI, a component of the ventral network) was significantly decreased in schizophrenic patients (66). In an N-back task, patients with schizophrenia had decreased inhibitory self-connections within the DAN regions, particularly in the left FEF and the left SPL (67). The interacting changes between the DAN and other networks have also been found. In schizophrenics during a working memory task, the DMN connectivity with the DAN was decreased (68, 69). In contrast to the default mode, patients demonstrated less connectivity in the executive control and dorsal attention networks (70).

Patients with TLE presented decreased functional connectivity in almost all of the regions within the DAN (31, 71). For example, the FC values of the bilateral frontal eye field (FEF) and left intraparietal sulcus (IPS) were decreased (72). Thus, the DAN can also be regarded as a biomarker to explain the common cognition pathological mechanisms between schizophrenia and TLE, for which neuroimaging studies have revealed similar connectivity changes. ECT has also been shown to influence the DAN in depressed patients (73). However, the influence of the DAN in schizophrenia requires further investigation.

## Central-Executive Networks

The CEN is a brain network that links the dorsal lateral prefrontal cortex (dlPFC) and posterior parietal cortex (pPC) (74) (Figure 1C). The CEN is frequently activated during typical fMRI executive tasks and its activity is often contrary to that of DMN activity (75). Brain imaging studies have shown that intelligence differences are positively correlated to functional interactions within the CEN, in both children and adults (76). Additionally, another important role of the CEN is to inhibit the DMN functions under certain conditions (77). This coordination between the CEN and the DMN is important in many neuropsychiatric disorders.

From recent literature, most results have reported that the CEN participates in a triple network including the DMN, the CEN, and the salience network (SN) rather than serving an isolated role in schizophrenia (78). Thus, we will be discuss this topic after introducing the SN.

## Salience Network

The SN is defined as a brain network comprised of the anterior insula (AI) and anterior cingulate cortex (ACC) (79) (Figure 1D). Physically, there is a strong functional connectivity within the SN, which is important for sensory perception and the coordination of behavioral responses (80, 81). Additionally, during many forms of emotional processing, the brain regions within the SN exhibit increased activity (82). The SN can also interact with the DMN and CEN to form a triple network, which participates in many mental process and disorders (83–85).

Schizophrenic patients show consistent abnormalities of insular signatures in both structural and functional neuroimaging studies, which indicates that the SN is involved in pathological processes (86). The SN has a direct correlation to schizophrenia symptoms, in which both auditory verbal hallucinations and delusions were detected to induce aberrant SN functional connectivity (87, 88). However, the strengths of these functional connectivities are not always homogeneous, because some studies have found a reduction in such connectivities (86), whereas other studies have reported a mixed pattern of increased and decreased connections (89). The interaction of the SN and DMN showed delayed communication that was directly correlated to positive and negative symptoms of schizophrenia (90). Additionally, a disrupted SN-CEN circuit also accounted for these schizophrenic symptoms (91). The triple network formed by SN-CEN-DMN nodes has shown dysregulated connections in schizophrenia (92) and mainly contributes to positive symptoms (93).

In TLE patients, one study reported that there was decreased connectivity to insula and ACC, suggesting a reduced SN (31). However, this result still needs to be further confirmed by more neuroimaging studies. When compared to schizophrenia, TLE showed a similar changes of the SN activity. However, it is difficult to confirm this hypothesis since there is still not enough neuroimaging evidence to properly assess this phenomenon. After ECT treatment, enhanced inter-network connectivity between the SN and the DMN has been found (73). However, whether this enhanced connectivity would be found in TLE patients requires further investigation.

## The Emerging Role of Graph Theory

An increasing number of studies has supported that schizophrenia and TLE are disorders involving abnormal brain networks rather than several abnormal discrete brain regions (29, 70). It may be difficult to reach a unifying result by analysis of regional activation/deactivation or connectivity abnormalities. Therefore, graph theory, which is a mathematical framework that allows for the quantitative modeling and analysis of networks, has been applied with increasing success to neuroimaging data (94). In graph theory, the brain can be represented as a graph, and the set of nodes may be composed of brain regions or voxels (on a macroscopic level) or individual neurons (on a cellular level). Thus, edges will represent the connections between these brain regions/voxels or individual neurons depending on the conditions (95). Then, this information can be encoded in a mathematical data structure called a connectivity matrix. Based on the connectivity matrix, graph theory provides a more large-scale assessment of the human brain and can provide more integrative information of various diseases (96).

The results of graph theoretical analysis on schizophrenia are still inconsistent, but there has been some convergence around the concept of topological randomization (97, 98). Previous studies have shown that the functional brain networks of schizophrenia are relatively shifted toward the randomness of small-world topology (99). Another study also confirmed that schizophrenics demonstrate significant randomization of global network metrics (100). Additionally, the parameters of the global-network topology of schizophrenia have been found to be decreased in both functional and anatomical networks (96, 101).

On the contrary, graph theoretical analyses of networks have provided evidence generally suggesting a shift toward a more regularized topology in TLE patients (102). Within the range of small-world topologies, a more regularized network topology is present in TLE patients (103). The increased path length and clustering in TLE patients supports this more regularized arrangement (104). Thus, interestingly, topological characteristics have revealed an antagonistic feature between schizophrenia and epilepsy, although these studies of schizophrenia and epilepsy were independently conducted.

However, there are limited reports regarding the effects of ECT on brain-network dynamics in schizophrenia. Whether ECT treatment reverses randomized brain networks to a more regularized pattern will require further investigations. As for the side effects of ECT, graph analysis of TLE patients showed that individuals with poor seizure control experienced more severe memory impairment (105). These results may indicate that a well-organized ECT practice may reduce side effects, but further neuroimaging evidence is still needed to validate or refute this hypothesis.

## Conclusions

Functional brain networks, such as the DMN, the SN and the DAN, have provided a new perspective to understand the relationship between schizophrenia and epilepsy. At this level, these two diseases show similar connectivity changes and suggest that they have more of an affinity-type relationship due to their similar pathological features. At larger scale, graph theoretical analysis has indicated an antagonistic relationship between these two diseases, although more evidence is needed to determine the validity of these findings. In addition, ECT treatment has been shown to modify the dynamics of these brain networks. If future studies verify that ECT treatment can reverse randomized brain networks in schizophrenia to more regularized patterns, the original premise for the creation of ECT may be further corroborated and better understood.

## Author Contributions

YX designed and supervised the study. QL and SL drafted the manuscript. MG and C-XY collected some literature.

### Conflict of Interest Statement

The authors declare that the research was conducted in the absence of any commercial or financial relationships that could be construed as a potential conflict of interest.
